# Insufficient Oral Behaviour and the High Need for Periodontal Treatment in Patients with Heart Insufficiency and after Heart Transplantation: A Need for Special Care Programs?

**DOI:** 10.3390/jcm8101668

**Published:** 2019-10-12

**Authors:** Christian Binner, Justus Wagner, Gerhard Schmalz, Mirjam Eisner, Josephine Rast, Tanja Kottmann, Rainer Haak, Andreas Oberbach, Michael A. Borger, Jens Garbade, Dirk Ziebolz

**Affiliations:** 1 Department of Cardiac Surgery, Leipzig Heart Center, University of Leipzig, 04289 Leipzig, Germany; Christian.Binner@leipzig-heart.de (C.B.); michael.borger@helios-gesundheit.de (M.A.B.); Jens.Garbade@medizin.uni-leipzig.de (J.G.); 2 Department of Cariology, Endodontology and Periodontology, University of Leipzig, 04103 Leipzig, Germany; justus7@gmx.de (J.W.); gerhard.schmalz@medizin.uni-leipzig.de (G.S.); mirjamcharlotte@web.de (M.E.); josephine.rast@medizin.uni-leipzig.de (J.R.); rainer.haak@medizin.uni-leipzig.de (R.H.); 3 CRO Dr. med. Kottmann GmbH & Co. KG, 59077 Hamm, Germany; tk@cro-kottmann.de; 4 Department of Diagnostics, Fraunhofer Institute for Cell Therapy and Immunology Leipzig, 04103 Leipzig, Germany; Andreas.Oberbach@medizin.uni-leipzig.de

**Keywords:** dental care, heart transplantation, oral health, dental behaviour

## Abstract

Background: The aim of this cross-sectional study was the assessment of dental behaviour and oral health condition of heart transplant recipients (HTx) in comparison to patients with heart insufficiency (HI). Methods: Patients attending the Department for Cardiac Surgery, Leipzig Heart Center, Germany were recruited. Standardized questionnaires regarding dental behaviour and periodontal complaints were applied. A dental (decayed-, missing- and filled-teeth index) and periodontal examination (periodontal probing depth (PPD) and clinical attachment loss (CAL)) was performed. Based on the oral findings, dental and periodontal treatment need was determined. Statistics: T-test, Mann-Whitney U test, Chi-square test, and Fisher-test (*p* < 0.05). Results: A total of 201 patients (HTx: 112, HI: 89) were included. HTx patients were significantly more often allocated to dentists (*p* < 0.01). Furthermore, the HTx patients rated feeling informed appropriately about oral health more often (*p* < 0.01). HTx patients used interdental cleaning (*p* < 0.01) and mouth rinse (*p* = 0.02) more often than HI patients. No differences between groups were present regarding dental status and periodontitis severity (*p* > 0.05). Periodontal treatment need was high, showing prevalence of 79.5% (HTx) and 87.6% (HI, *p* = 0.14), respectively. Conclusions: Both groups show insufficient oral behaviour and a high need for periodontal treatment. Special care programs for HTx candidates and recipients appear recommendable.

## 1. Introduction

Heart transplantation has developed into preferred therapy for selected patients with end-stage heart failure. Besides the increasing success of this therapy, long-term complications are still a very important issue [1]. Thereby, infectious complications, renal failure, and malignancy frequently occur as a consequence of their necessary life-long immunosuppression [1,2]. The reduction of these long-term complications of immunosuppressive therapy by different strategies seems an important issue to improve individual care of heart transplant recipients [2,3]. 

The oral cavity is an important habitat for bacteria and simultaneously an important source for bacteria which enter the systemic circulation of patients [4]. These bacteria have the potential to cause serious infectious complications in immunocompromised individuals [5]. Accordingly, patients should receive a comprehensive dental rehabilitation prior to transplantation, including periodontal and dental therapy, to minimize the risk of systemic infections in these vulnerable patient groups [6]. Thereby, the adequate assessment and care by a qualified specialist and application of a structured post-transplant follow-up program seems recommendable to avoid oral disease-related systemic complications in transplant recipients [6]. In contrast to these recommendations, patients before and after organ transplantation (kidney, liver, lung) were reported to show an insufficient oral status, especially with a high prevalence of periodontal disease [7,8,9,10]. Moreover, transplant recipients in different German transplant centres were found to present a high dental and especially periodontal treatment need, irrespectively of the time since transplantation [11]. This indicates that recently the upper mentioned recommendations would be not fulfilled. Data for heart transplant recipients are still rare in the literature. A recent Chinese cross-sectional study presented worse periodontal conditions for heart transplant recipients [12]. It was also shown that periodontal inflammation would be associated with systemic inflammatory parameters in heart transplanted individuals [13]. However, data regarding the state of dental care of heart transplant recipients as well as their oral behaviour, especially compared to patients suffering from heart insufficiency in Germany, are not available yet. These insights might be helpful to detect current deficits in dental care of these patients and might deliver potential approaches to improve this situation. 

Therefore, the current study aimed to assess dental behaviour, periodontal complaints, and the dental and periodontal condition of heart transplant recipients (HTx) in comparison to patients suffering from heart insufficiency (HI). Moreover, potential associations between periodontal complaints and clinically assessed treatment need should be investigated to estimate the ability to apply such a questionnaire-based screening in care of the patients. It was hypothesised that oral behaviour as well as dental and periodontal conditions would be insufficient in both HTx recipients and patients with HI. 

## 2. Methods

This study was designed as a cross-sectional study to assess and compare the dental behaviour as well as oral health parameters of patients with heart insufficiency (HI) and after heart transplantation (HTx). The study has been reviewed and approved by the ethics committee of the Medical Faculty of University of Leipzig (No: 414/16-ek). All included participants were informed verbally and in writing and provided their written informed consent.

### 2.1. Patients

Patients attending the University Department for Cardiac Surgery, Heart Center Leipzig, Germany for their regular follow-up appointments were informed about aims and course of the investigation and asked for their voluntarily participation in the current study. Mandatory criteria for inclusion were the ability to provide informed consent and age of at least 18 years. The following exclusion criteria were defined:

- Clinical examination impossible due to worse general health status;
- Autoimmune diseases (e.g., rheumatoid arthritis, chronic inflammatory bowel diseases);
- Infectious diseases including hepatitis A, B, C, tuberculosis, or HIV;
- Addiction (alcohol or drugs);
- Pregnancy.

Patients who fitted into the in- and exclusion criteria and provided informed consent were allocated to the questionnaire-based and oral examination. Furthermore, general and clinical data were extracted from medical history of the participants. Thereby, smoking habits (smoker: currently smoking, former smoker: smoking within five years before examination, nonsmoker: no smoking for at least five years), underlying heart diseases, comorbidities, relevant medications, and current blood parameters were extracted, if applicable. 

### 2.2. Questionnaires

Two different standardized questionnaires were applied to the included participants. On the one hand, a periodontitis questionnaire, which assessed periodontitis-related complaints, was used as described previously [14]. On the other hand, a specific questionnaire, used in previous studies of this working group, regarding dental behaviour, including utilization of dental offers, oral hygiene procedures as well as disease related issues was used [8,9,10].

### 2.3. Oral Examination 

The oral examination was performed once under standardized conditions at the Department of Cardiothoracic Surgery, Leipzig Heart Centre, Leipzig, Germany. The dental and periodontal investigation was executed by two experienced and calibrated dentists (kappa > 0.8). Prior to dental and periodontal examination, participants received an antibiotic prophylaxis (2 g Amoxicillin) or clindamycine according to the recent guidelines [15]. 

At first an examination of the oral mucosa was performed to detect gingival overgrowth or any kind of changes of oral mucosa. The dental investigation comprised the assessment of teeth showing a carious cavitation of the tooth surface (D-T), missing teeth (M-T) as well as teeth restored with a filling or crown (F-T). According to WHO, the DMF-T index was assessed [16]. Based on the presence of carious lesions deserving dental intervention (D-T > 0), dental treatment need was defined. 

The periodontal examination included the evaluation of periodontal probing depth (PPD) and clinical attachment loss (CAL) with a periodontal probe (PCP 15, Hu-Friedy, Chicago, IL, USA). Based on these parameters, periodontitis severity according to Eke et al. 2012 was categorized into no/mild, moderate, or severe periodontitis [17]. Furthermore, the presence of PPD ≥ 3.5 mm in at least two different sextants was defined as periodontal treatment need in accordance to periodontal-screening index (PSI) [18,19]. Patients showing dental and/or periodontal treatment needs were classified as having “overall treatment need”.

### 2.4. Statistical Analysis 

The statistical analysis was performed with SPSS for Windows, version 24.0 (SPSS Inc., Chicago, Illinois, USA). The metric variables were tested for their normal distribution with Kolmogorov–Smirnov test. For comparison of two independent, normal distributed samples, a T-test was applied, after testing for homogeneity of variances with Levene test. In case of homogeneity of variances, Student’s t-test was used. In case of non-normal distribution, Mann-Whitney U test was applied. The categorical data were analysed with Chi-square or Fisher-test, respectively. For all applied analyses, a two-sided significance testing was used, whereby the significance level has been set at *p* < 0.05.

## 3. Results

### 3.1. Patients

A total of 201 patients were included in the current study, from which 112 participants were after HTx (mean age: 56.15 ± 12.23 years, average time since HTx: 7.1 ± 5.50 years) and 89 were HI patients (mean age 55.47 ± 10.91 years; *p* = 0.22). In the HI group, more patients were smokers compared to HTx group (*p* = 0.02). Regarding underlying heart disease, co-morbidities, medication, and blood concentration of leucocytes and c-reactive protein (CRP), several differences were found between HTx and HI group (*p*_i_ ≤ 0.01; Table 1).

### 3.2. Questionnaires

The results of the dental behaviour and periodontitis questionnaires are presented in Table 2 and Table 3. HTx patients were significantly more often allocated to dentists than the HI group (88.1% vs. 41.5%; *p <* 0.01). Furthermore, HTx patients rated dentists’ knowledge about underlying disease and their information about antibiotic prophylaxis higher than the HI group (*p <* 0.01). Additionally, HTx patients stated more often to feel informed appropriately than HI patients (88.8% vs. 72.4%, *p <* 0.01). Regarding usage of oral hygiene aids, HTx used interdental cleaning (33.9% vs. 15.7%, *p* < 0.01) and mouth rinse (62.5% vs. 44.9%, *p* = 0.02) more often than the HI group (Table 2). In the periodontitis questionnaire, HTx patients reported more frequently to suffer from tooth pain (17.1% vs. 3.6%, *p <* 0.01) and more compliant-oriented visits to the dentist (25.7% vs. 11.9%, *p* = 0.02; Table 3).

### 3.3. Oral Examination

The results of oral examination are given in Table 4. No differences between both groups were present regarding dental status (DMF-T) and periodontitis severity (*p >* 0.05). The dental treatment need was approximately low and comparable between HTx and HI (15.2% vs. 15.7%, *p* = 0.99). The periodontal treatment need was high, showing prevalence of 79.5% (HTx) and 87.6% (HI, *p* = 0.14), respectively (Table 4).

Furthermore, the presence of swollen gingiva (*p* = 0.03), bleeding gums (*p* < 0.01), and the attendance to regular professional tooth cleaning (*p* < 0.01) in the periodontitis questionnaire was associated with periodontal treatment need in the total cohort (Table 5).

## 4. Discussion

Summary of the main results: HTx patients were found to present a slightly improved dental behaviour compared to the HI group. However, HTx recipients reported little more complaints and complaint-oriented dentist consultations in the periodontitis questionnaire. Especially, the periodontal treatment need was high for both groups, whereby oral health status was comparable between HTx and HI group. Considering the total cohort, the presence of swollen and bleeding gums rated in the questionnaire showed an association with periodontal treatment need. 

Comparison with the literature: The current study did not include a healthy control group. As performed previously [8,9,10], the oral conditions in general can be interpreted considering the results of the Fifth German Oral Health Study (DMS V), a representative study for the German general population [20]. While the mean age of participants in the current study was on average approximately 56 years, this age group is not exclusively reflected in the DMS V. The slightly higher age group of 65–74 years in the DMS V showed a DMF-T of 17.7, a D-T of 0.5, and an M-T of 11.1, which is almost comparable to the current study’s findings (Table 6) [20]. Accordingly, a comparable dental status to healthy general population can be assumed for both HI and HTx patients. The prevalence of 64.4% of moderate to severe periodontitis reported in DMS V appears slightly lower than for the current study´s participants. Similarly, considering a periodontal treatment need of 75.4% of general population (DMS V), this seems also slightly higher in the both groups within the current study. Accordingly, a high periodontal treatment need of patients with HI and after HTx, which is almost higher than for the German general population in the DMS V [20] can be concluded. This result is in line with a recent Chinese cross-sectional study, which presented worse periodontal conditions for HTx recipients, while dental care status was comparable to healthy controls [12]. Similarly, patients who received a solid organ transplantation (kidney, liver, lung) were found to present high periodontal treatment need, irrespective of time since transplantation [7,8,9,10,11]. Different explanations might be applicable for this situation for the patients in the current study. On the one hand, HI and HTx might influence the periodontitis pathogenesis in the affected patients. Schulze-Späte et al. presented an increase in turnover markers of the alveolar bone of patients with heart insufficiency compared to healthy controls [21]. Furthermore, there are associations between periodontitis and coronary heart diseases, which are already well documented in the literature [22]. Besides inflammatory effects and a role of potentially periodontal bacteria, a shared risk complex, including smoking and metabolic disorders could link heart diseases and periodontitis [22,23]. The importance of these factors for the current study´s findings are difficult to estimate. 

The more relevant point might be the dental care situation of patients with HI and after HTx. While majority of HTx recipients stated that they have been allocated to a dentist prior to HTx, only the vast minority have received periodontal therapy from their dentist. This might indicate a gap in dental care, regarding periodontal rehabilitation of HTx candidates and recipients. This situation is critical, because periodontal inflammation leads to increased permeability of periodontal tissue [24], which is associated with an increased risk for bacteraemia caused by chewing, oral hygiene measures, and dental therapy procedures [25,26]. Transplant recipients are under a lifelong immunosuppressive medication with an increased risk of systemic infections [1,2]; thus, the high periodontal treatment need might be a serious risk for their systemic health. 

The HTx recipients within the current study were found to present a slightly improved dental behaviour. Thereby, the HTx group appears to be informed well about antibiotic prophylaxis and subjectively feel informed better than HI group. Furthermore, the usage of aids for interdental cleaning was higher in the HTx than in the HI group, although it was still approximately low. A similar tendency was previously found for kidney and liver transplanted individuals, respectively [8,9]. The low usage of interdental cleaning aids might be a further reason for the high periodontal treatment need, as interdental cleaning devices reduce inflammation more effectively than brushing alone [27]. Moreover, only a minority of patients used a power toothbrush, although this would be recommendable for periodontally diseased patients [28]. In summary, although HTx showed slightly better oral behaviour than the HI group, a lack in appropriate information and/or patient motivation must be assumed. In combination with the high treatment need, the demand of special care programs, which is already pointed to in the literature [6], can be supported by the current study.

Another aspect was the applied periodontitis questionnaire. This validated tool can reflect the presence of periodontal complaints and inflammation [14]. For the total cohort, bleeding and swollen gums were associated with the presence of periodontal treatment need. The absence of further associations might be caused by the generally high prevalence of periodontal treatment need. However, and regardless, transplant centres might use these questions about bleeding and swelling of the gums for their patients to detect high-risk patients with the necessity of allocation to the dentist. 

Strengths and limitations: This current study is the first and largest examination of patients with HI and after HTx in Germany. The standardized examination and application of valid questionnaires strengthens the findings. Furthermore, the focus on treatment need and the potential application of periodontal complaint-related questions for HTx recipients are an approach with clinical potential. However, there are several limitations to address. The cross-sectional design limits the ability to draw any causative conclusions. The absence of a healthy control group must also be mentioned, although the comparison to representative study is a repeatedly applied and functioning concept [7,8,9,10]. Furthermore, the occurrence of infectious complications due to the high periodontal treatment need stays unclear. The analysis of blood values related to periodontal treatment need would be an interesting approach, as a recent study found the CRP to be related to periodontal inflammation [13]. Nevertheless, this was beyond the focus of the current study, which aimed at the assessment of the recent care situation of HTx and HI patients. A further influential factor that has to be considered in the interpretation of periodontal findings is the difference in smoking habits, comorbidities, and medication between HI and HTx. A matching would have been helpful; however, it was aimed to include as many patients as possible. Moreover, the assessment of periodontitis is limited by the fact that the new classification for periodontal and peri-implant diseases and conditions with a staging and grading matrix [29] was not applied. The usage of this recent classification scheme is recommended for scientific evaluation of periodontitis and should be applied accordingly [29]. However, at the time when the current study was planned and executed, this new classification was not available. For appropriate staging and grading, many diagnostic findings (e.g., furcation, tooth mobility, radiographic bone loss) as well as rate of progression are needed. Therefore, these necessary data have not been recorded completely and progression rate was not detectable within a cross-sectional study. Thus, the periodontitis severity was determined according to Eke et al. 2012 [17], which is validated even in cohorts of patients with general diseases [8,9,10] and used in the DMS V as a reference for German general population [20]. 

## 5. Conclusions 

Patients with HI and after HTx show a high periodontal treatment need. While HTx recipients show a slightly improved oral behaviour, a lack in information can be assumed. A special care program for HTx candidates and recipients appears necessary and should include early pretransplant rehabilitation and sufficient maintenance after HTx. 

## Figures and Tables

**Table 1:** Patient characteristics, significance level: *p* < 0.05.

		HTx (*n* = 112)	HI (*n* = 89)	*p*-Value
Gender (female in % (*n*))		23.2% (26)	15.7% (4)	0.22
Age in years (mv ± sd)		56.15 ± 12.23	55.47 ± 10.91	0.57
Time since HTx in years (mv ± sd)		7.1 ± 5.50	-	-
Smoking habits % (*n*)	smoker	5.4% (6)	17% (15)	**0.02**
	nonsmoker	74.1% (83)	60.3% (54)	
	former smoker	20.5% (23)	22.7% (20)	
Ejection fraction in % (mv ± sd)		57.31 ± 8.04	28.17 ± 9.36	<0.01
Underlying heart disease % (*n*)	DCM	61.6% (69)	61.8% (55)	0.99
	ICM	31.3% (35)	32.6% (29)	0.88
	valvular insufficiency	13.4% (15)	38.2% (34)	**<0.01**
	atrial fibrillation	6.3% (7)	29.2% (26)	**<0.01**
Co-morbidities % (*n*)	hypertension	55.9% (62)	74.2% (66)	**0.01**
	diabetes mellitus	34.8% (39)	36% (32)	0.88
	osteoporosis	5.4% (6)	4.5% (4)	0.99
	renal insufficiency	84.8% (95)	44.9% (40)	**<0.01**
	adipositas	51.8% (58)	42.7% (38)	0.21
Medication relevant to oral conditions % (*n*)	calcium-channel blocker	25.2% (28)	5.7% (5)	**<0.01**
	immunosuppression	100% (112)	5.7% (5)	**<0.01**
	bisphopshonates	31.8% (35)	4.6% (4)	**<0.01**
Blood parameters (mv ± sd) (at the time of evaluation)	CRP (mg/L)	5.40 ± 10.49	11.65 ± 23.57	**0.03**
	creatinine (µmol/L)	45.88 ± 111.78	45.95 ± 73.68	0.67
	hemoglobin (mmol/ L)	8.55 ± 1.88	8.23 ± 1.76	0.68
	erythrocytes (10^6^/µ L)	4.53 ± 0.62	4.61 ± 0.74	0.56
	leukocytes (10³/µ L)	6.50 ± 1.90	7.79 ± 2.76	**0.01**
	platelet (10³/µ L)	214.73 ± 77.88	237.93 ± 76.33	0.07
NYHA class	I	-	5% (4/76)	-
	I–II		5% (4/76)	
	II		47% (36/76)	
	II–III		5% (4/76)	
	III		26% (4/76)	
	III–IV		5% (4/76)	
	IV		5% (4/76)	

HTx: heart transplantation, HI: heart insufficiency, CRP: c-reactive protein, mv: mean value, sd: standard deviation, DCM: dilatative cardio-myopathy, ICM: ischeamic cardio-myopathy; significant values (*p <* 0.05) are highlighted in bold.

**Table 2:** Results of the dental behaviour between groups, significance level: *p <* 0.05.

		HTx	HI	*p*-Value
Allocation to dentist % (*n*)		88.1% (96/109)	41.5% (34/82)	**<0.01**
Dental visit before Tx % (*n*)		82.7% (91/110)	-	-
Dental treatment before Tx % (*n*)	tooth extraction	26.8% (30/112)	-	-
	restorative treatment	55.4% (62/112)		
	periodontal therapy	6.3% (7)		
	Control/no invasive treatment	25% (28/112)		
Regular dental treatment % (*n*)		67% (73/109)	77% (67/87)	0.15
Dentists knowledge about underlying disease % (*n*)		90.9% (100/110)	56.6% (47/83)	**<0.01**
Information about antibiotic prophylaxis % (*n*)		74.8% (83/111)	37% (30/81)	**<0.01**
Information about relationship oral-general health % (*n*)		13.4% (15/112)	6.7% (6/89)	0.17
Feel informed appropriately		88.8% (95/107)	72.4% (63/87)	**0.01**
last dental examination % (*n*)	0–3 months	33.9% (38/112)	27.6% (24/87)	0.69
	3–12 months	52.7% (59/112)	57.5% (50/87)	
	>12 months	13.4% (15/112)	14.9% (13/87)	
oral hygiene: tooth brushing % (*n*)	<1x/day	1.8% (2/111)	4.7% (4/86)	0.07
	1–2x/day	75.7% (84/111)	83.7% (72/86)	
	>2x/day	22.5% (25/111)	11.6% (10/86)	
oral hygiene aids % (*n*)	manual toothbrush	64.3% (72/112)	74.2% (66/89)	0.17
	power toothbrush	38.4% (43/112)	30.3% (27/89)	0.30
	dental floss/interdental brush	33.9% (38/112)	15.7% (14/89)	**<0.01**
	mouth rinse	62.5% (70/112)	44.9% (40/89)	**0.02**
	fluoride gel	6.3% (7/112)	11.2% (10/89)	0.31

HTx: heart transplantation, HI: heart insufficiency; significant values (*p <* 0.05) are highlighted in bold.

**Table 3:** Results of the periodontitis questionnaire, significance level: *p <* 0.05.

Symptoms	HTx	HI	*p*-Value
swollen gingiva % (*n*)	13.4% (15/112)	6.9% (6/87)	0.17
aching gums % (*n*)	11.6% (13/112)	14.9% (13/87)	0.53
sensitive gums % (*n*)	25.2% (28/111)	20.7% (18/87)	0.50
bleeding gums % (*n*)	20% (22/110)	28.2% (24/85)	0.23
Recession % (*n*)	30.9% (34/119)	26.7% (23/86)	0.64
tooth hypersensitivity % (*n*)	28.8% (32/111)	31% (27/87)	0.76
tooth mobility % (*n*)	9.9% (11/111)	8.1% (7/86)	0.81
tooth migration % (*n*)	9.1% (10/110)	7.1% (6/85)	0.79
change in occlusion % (*n*)	7.2% (8/111)	3.6% (3/84)	0.36
halitosis % (*n*)	17.3% (19/110)	21.2% (18/85)	0.58
bad taste % (*n*)	16.2% (18/111)	16.5% (14/85)	0.99
tooth pain % (*n*)	17.1% (19/111)	3.6% (3/83)	**<0.01**
previous periodontitis therapy % (*n*)	15.5% (17/110)	15.5% (13/84)	0.99
regular control investigation % (*n*)	77.7% (87/112)	77% (67/87)	0.99
Complaint-oriented investigation % (*n*)	25.7% (28/109)	11.9% (10/84)	**0.02**
regular professional tooth cleaning % (*n*)	39.6% (44/111)	44.7% (38/85)	0.56

HTx: heart transplantation, HI: heart insufficiency; significant values (*p <* 0.05) are highlighted in bold.

**Table 4:** Oral health situation between the two groups, significance level: *p <* 0.05.

Parameter		HTx (*n* = 112)	HI (*n* = 89)	*p*-Value
DMF-T (mv ± sd)		16.93 ± 7.52	17.78 ± 7.06	0.46
D-T (mv ± sd)		0.26 ± 0.74	0.28 ± 0.75	0.88
M-T (mv ± sd)		8.41 ± 8.88	8.94 ± 9.22	0.68
F-T (mv ± sd)		8.26 ± 5.54	8.55 ± 5.66	0.75
Gingival overgrowth % (*n*)		6% (7)	0	**<0.01**
Perio-dontitis % (*n*)	no/mild	21.4% (24)	22.5% (20)	0.28
	moderate	50.9% (57)	40.4% (36)	
	severe	27.7% (31)	37.1% (33)	
dental treatment need % (*n*)		15.2% (17)	15.7% (14)	0.99
periodontal treatment need % (*n*)		79.5% (89)	87.6% (78)	0.14
overall treatment need % (*n*)		80.4% (90)	87.6% (78)	0.18

HTx: heart transplantation, HI: heart insufficiency, mv: mean value, sd: standard deviation; significant values (*p <* 0.05) are highlighted in bold.

**Table 5:** Association between periodontitis questionnaire and periodontal treatment need within the total cohort; significance level: *p <* 0.05.

Symptoms	*p*-Value
swollen gingiva	**0.03**
aching gums	0.99
sensitive gums	0.12
bleeding gums	**<0.01**
recession	0.15
tooth hypersensitivity	0.42
tooth mobility	0.32
tooth migration	0.99
change in occlusion	0.99
halitosis	0.46
bad taste	0.12
tooth pain	0.77
previous periodontitis therapy	0.43
regular control investigation	0.26
Complaint-oriented investigation	0.63
regular professional tooth cleaning	**<0.01**

Significant values (*p* < 0.05) are highlighted in bold.

**Table 6:** Comparison of dental findings with the Fifth German Oral Health Study (DMS V) (Jordan et al. 2016).

Parameter		HTx (*n* = 112)	HI (*n* = 89)	DMS V Age Group 35–44 Years	DMS V Age Group 65–74 Years
DMF-T (mv ± sd)		16.9 ± 7.5	17.8 ± 7.1	11.2	17.7
D-T (mv ± sd)		0.3 ± 0.7	0.3 ± 0.8	0.5	0.5
M-T (mv ± sd)		8.4 ± 8.9	8.9 ± 9.2	2.1	11.1
F-T (mv ± sd)		8.3 ± 5.5	8.6 ± 5.7	8.6	6.1
Periodontitis % (*n*)	no/mild	21%	23%	48%	35%
	moderate	51%	40%	43%	45%
	severe	28%	37%	8%	20%

HTx: heart transplantation, HI: heart insufficiency, mv: mean value, sd: standard deviation.
